# Supplementary material for: Metabolomic profiles of sleep-disordered breathing are associated with hypertension and diabetes mellitus development
Source: Nat Commun. 2024 Feb 28;15:1845. doi: 10.1038/s41467-024-46019-y (PMC10902315; doi:10.1038/s41467-024-46019-y)
Supplement: Supplementary file 1 — Supplementary Information [file 41467_2024_46019_MOESM1_ESM.pdf]

Metabolomic Profiles of Sleep-Disordered Breathing are Associated with Hypertension and Diabetes Mellitus Development: Supplementary Information

Contents

Supplementary Note 1 1

Supplementary Figure S1. Scree plot for Principal Component Analysis (PCA) on the sleep disordered breathing (SDB) phenotypes in HCHS/SOL..3

Supplementary Figure S2. Comparison of progesterone steroids metabolites levels in the combined batch 4

Supplementary Figure S3. Single metabolite association analysis in both gender in batch 1 model 1.................6

Supplementary Figure S4. Super pathway breakdown for metabolites selected by LASSO for SDB PCs..............7

Supplementary Figure S5. The associations between SDB metabolomic indices in quartiles and incident cardiometabolic outcomes in the combined batch in model1..8

Supplementary Figure S6. Study sample selection for the metabolomic analysis 9

References..10

Supplementary Note 1

Sample Preparation: Samples were prepared using the automated MicroLab STAR® system from Hamilton Company. Several recovery standards were added prior to the first step in the extraction process for QC purposes. To remove protein, dissociate small molecules bound to protein or trapped in the precipitated protein matrix, and to recover chemically diverse metabolites, proteins were precipitated with methanol under vigorous shaking for 2 min (Glen Mills GenoGrinder 2000) followed by centrifugation. The resulting extract was divided into five fractions: two for analysis by two separate reverse phase (RP)/UPLC-MS/MS methods with positive ion mode electrospray ionization (ESI), one for analysis by RP/UPLC-MS/MS with negative ion mode ESI, one for analysis by HILIC/UPLC-MS/MS with negative ion mode ESI, and one sample was reserved for backup. Samples were placed briefly on a TurboVap® (Zymark) to remove the organic solvent. The sample extracts were stored overnight under nitrogen before preparation for analysis.

Ultrahigh Performance Liquid Chromatography-Tandem Mass Spectroscopy (UPLC-MS/MS): All methods utilized a Waters ACQUITY ultra-performance liquid chromatography (UPLC) and a Thermo Scientific Q-Exactive high resolution/accurate mass spectrometer interfaced with a heated electrospray ionization (HESI-II) source and Orbitrap mass analyzer operated at 35,000 mass resolution. The sample extract was dried then reconstituted in solvents compatible to each of the four methods. Each reconstitution solvent contained a series of standards at fixed concentrations to ensure injection and chromatographic consistency. One aliquot was analyzed using acidic positive ion conditions, chromatographically optimized for more hydrophilic compounds. In this method, the extract was gradient eluted from a C18 column (Waters UPLC BEH C18-2.1x100 mm, 1.7 µm) using water and methanol, containing 0.05% perfluoropentanoic acid (PFPA) and 0.1% formic acid (FA). Another aliquot was also analyzed using acidic positive ion conditions, however it was chromatographically optimized for more hydrophobic compounds. In this method, the extract was gradient eluted from the same afore mentioned C18 column using methanol, acetonitrile, water, 0.05% PFPA and

0.01% FA and was operated at an overall higher organic content. Another aliquot was analyzed using basic negative ion optimized conditions using a separate dedicated C18 column. The basic extracts were gradient eluted from the column using methanol and water, however with 6.5mM Ammonium Bicarbonate at pH 8. The fourth aliquot was analyzed via negative ionization following elution from a HILIC column (Waters UPLC BEH Amide 2.1x150 mm, 1.7 µm) using a gradient consisting of water and acetonitrile with 10mM Ammonium Formate, pH 10.8. The MS analysis alternated between MS and data-dependent MS^n scans using dynamic exclusion. The scan range varied slighted between methods but covered 70-1000 m/z. Raw data files are archived and extracted as described below.

Data Extraction and Compound Identification: Raw data was extracted, peak-identified and QC processed using Metabolon's hardware and software. These systems are built on a web-service platform utilizing Microsoft's .NET technologies, which run on high-performance application servers and fiber-channel storage arrays in clusters to provide active failover and load-balancing. Compounds were identified by comparison to library entries of purified standards or recurrent unknown entities. Metabolon maintains a library based on authenticated standards that contains the retention time/index (RI), mass to charge ratio (*m/z)*, and chromatographic data (including MS/MS spectral data) on all molecules present in the library. Furthermore, biochemical identifications are based on three criteria: retention index within a narrow RI window of the proposed identification, accurate mass match to the library +/- 10 ppm, and the MS/MS forward and reverse scores between the experimental data and authentic standards. The MS/MS scores are based on a comparison of the ions present in the experimental spectrum to the ions present in the library spectrum. While there may be similarities between these molecules based on one of these factors, the use of all three data points can be utilized to distinguish and differentiate biochemicals. More than 3300 commercially available purified standard compounds have been acquired and registered into LIMS for analysis on all platforms for determination of their analytical characteristics. Additional mass spectral entries have been created for structurally unnamed biochemicals, which have been identified by virtue of their recurrent nature (both chromatographic and mass spectral). These compounds have the potential to be identified by future acquisition of a matching purified standard or by classical structural analysis.

Metabolite Quantification and Data Normalization: Peaks were quantified using area-under-the-curve. For studies spanning multiple days, a data normalization step was performed to correct variation resulting from instrument inter-day tuning differences. Essentially, each compound was corrected in run-day blocks by registering the medians to equal one (1.00) and normalizing each data point proportionately.

Supplementary Figure S1. Scree plot for Principal Component Analysis (PCA) on the sleep disordered breathing (SDB) phenotypes in HCHS/SOL

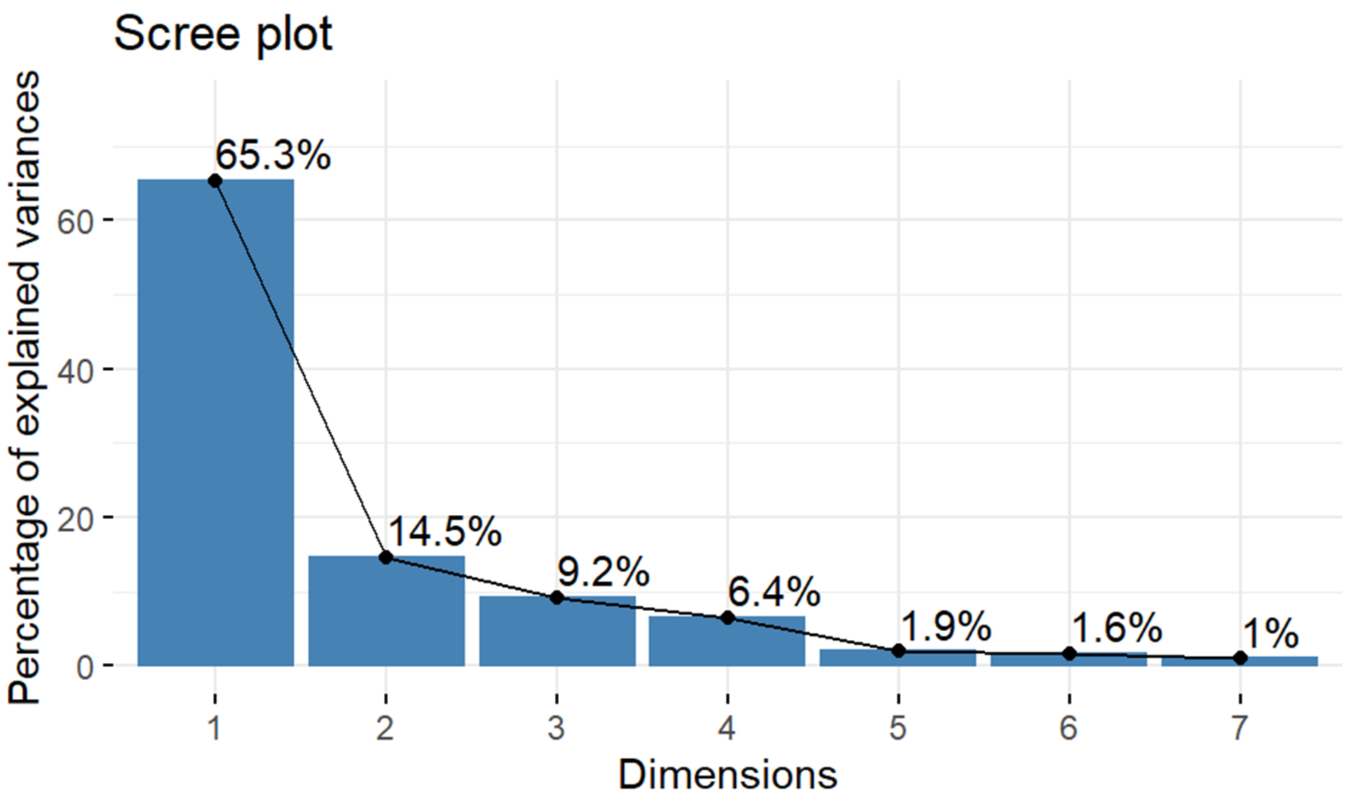

PCA was implemented on the following SDB phenotypes: REI3: Respiratory Event Index (REI) computed over all respiratory events, defined as apneas or hypopneas with at least 50% cannula flow reduction for a minimum duration of 10 seconds with >=3% oxygen desaturation; REI0: REI computed over all respiratory events regardless of oxygen desaturation; HB: hypoxic burden; avgEventLength: the average length of apnea and hypopnea events (combined); minSpO2: minimum oxyhemoglobin saturation during sleep; avgSpO2: average oxyhemoglobin saturation during sleep; Per90: percentage of sleep time with oxyhemoglobin saturation below 90%.

Supplementary Figure S2. Comparison of progesterone steroids metabolites levels in the combined batch

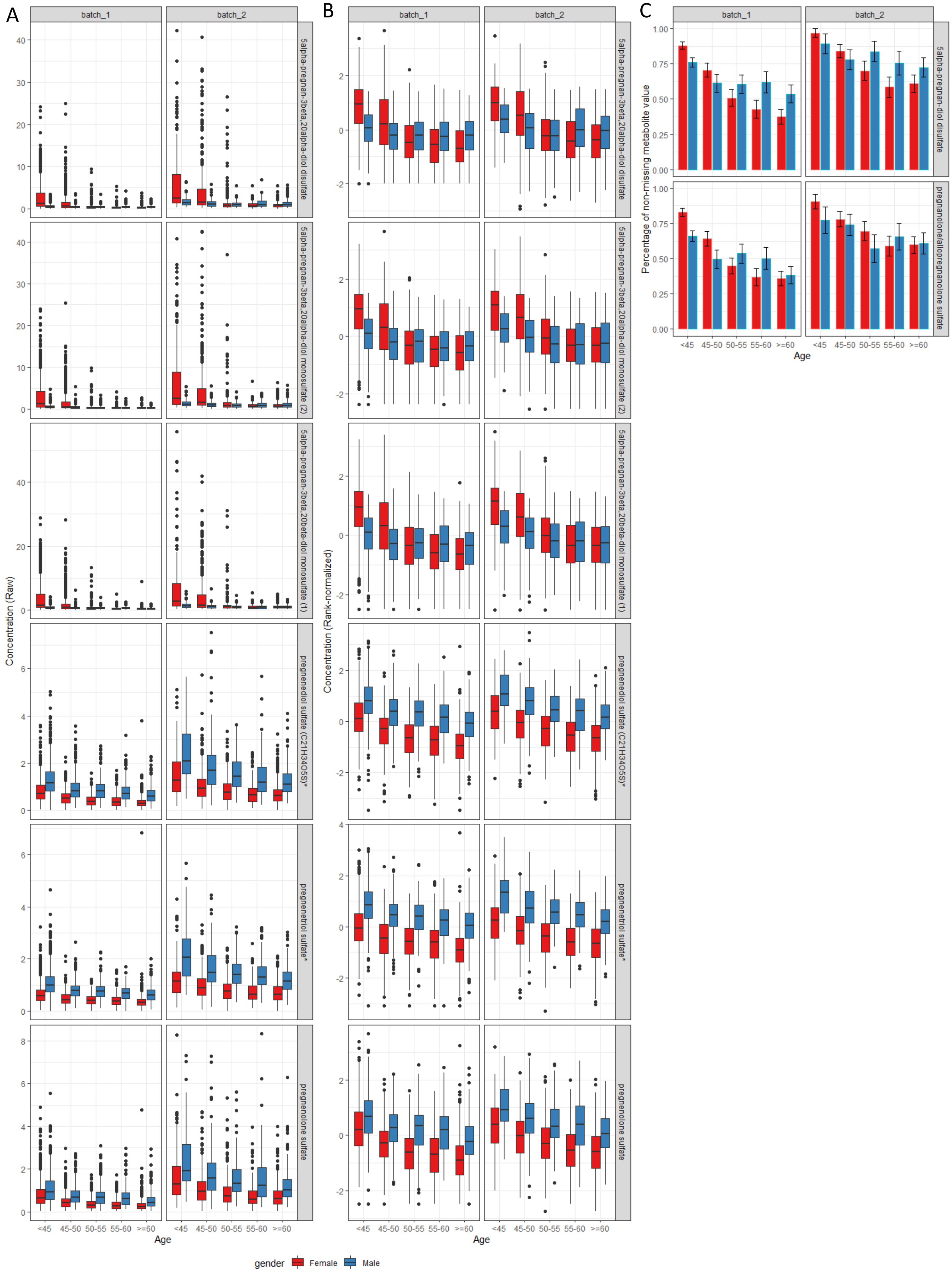

Panel A: Boxplot of raw concentration level of selected progesterone steroid sulfate metabolites in each sex stratum by age group in the combined batch (batch 1 and 2). Panel B: Boxplot of rank-normalized selected progesterone steroid sulfate metabolites in each sex stratum by age group in the combined batch (batch 1 and 2). The rank normalization was carried out in both sexes combined. Panel C: The percentage of study samples with non-missing value in each sex stratum by age group.

Metabolite with * indicates they were identified based on accurate mass data, retention time and mass spectrometry but not reference standards. Therefore, the verification is not as robust as metabolites without *.

Supplementary Figure S3. Single metabolite association analysis in both gender in batch 1 model 1

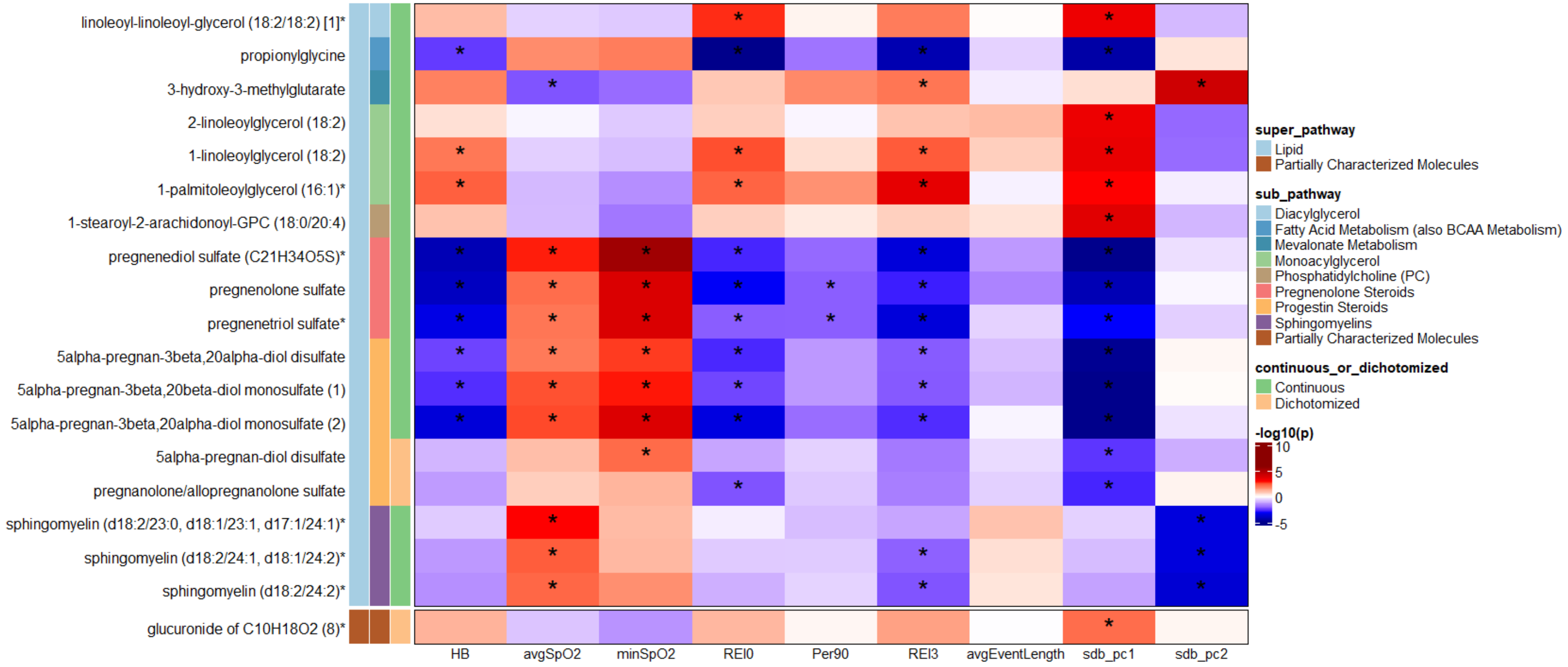

-log10(*p*) is based on unadjusted two-sided *p* derived by accounting for the complex sampling design-based degrees of freedom, using adjusted standard errors to compute the t-statistic in single metabolite association analysis with corresponding SDB phenotypes as dependent variables. * indicates FDR-corrected *p*, derived using the Benjamini-Hochberg method to control false discovery rate (FDR) for multiple testing among metabolites in all models for each SDB PC in the discovery dataset (batch 1) is below 0.05 for sdb_pc1 and sdb_pc2, and indicates unadjusted *p*<0.01 for the remaining 7 SDB phenotypes.

sdb_pc1: the first principal component of the 7 sleep disordered breathing traits included in the principal component analysis; sdb_pc2: the second principal component of the seven sleep disordered breathing traits included in the principal component analysis. REI3: Respiratory Event Index (REI) computed over all respiratory events, defined as apneas or hypopneas with at least 50% cannula flow reduction for a minimum duration of 10 seconds with >=3% oxygen desaturation; REI0: REI computed over all respiratory events regardless of oxygen desaturation; HB: hypoxic burden; avgEventLength: the average length of apnea and hypopnea events (combined); minSpO2: minimum oxyhemoglobin saturation during sleep; avgSpO2: average oxyhemoglobin saturation during sleep; Per90: percentage of sleep time with oxyhemoglobin saturation below 90%;

Model 1 adjusted for demographic variables, including age, sex, field center, Hispanic/Latino background (Mexicans, Puerto Ricans, Cubans, Central Americans, Dominicans, and South Americans and other/multi) and body mass index (BMI). Metabolite with * indicates they were identified based on accurate mass data, retention time and mass spectrometry but not reference standards. Therefore, the verification is not as robust as metabolites without *

Supplementary Figure S4. Super pathway breakdown for metabolites selected by LASSO for SDB PCs

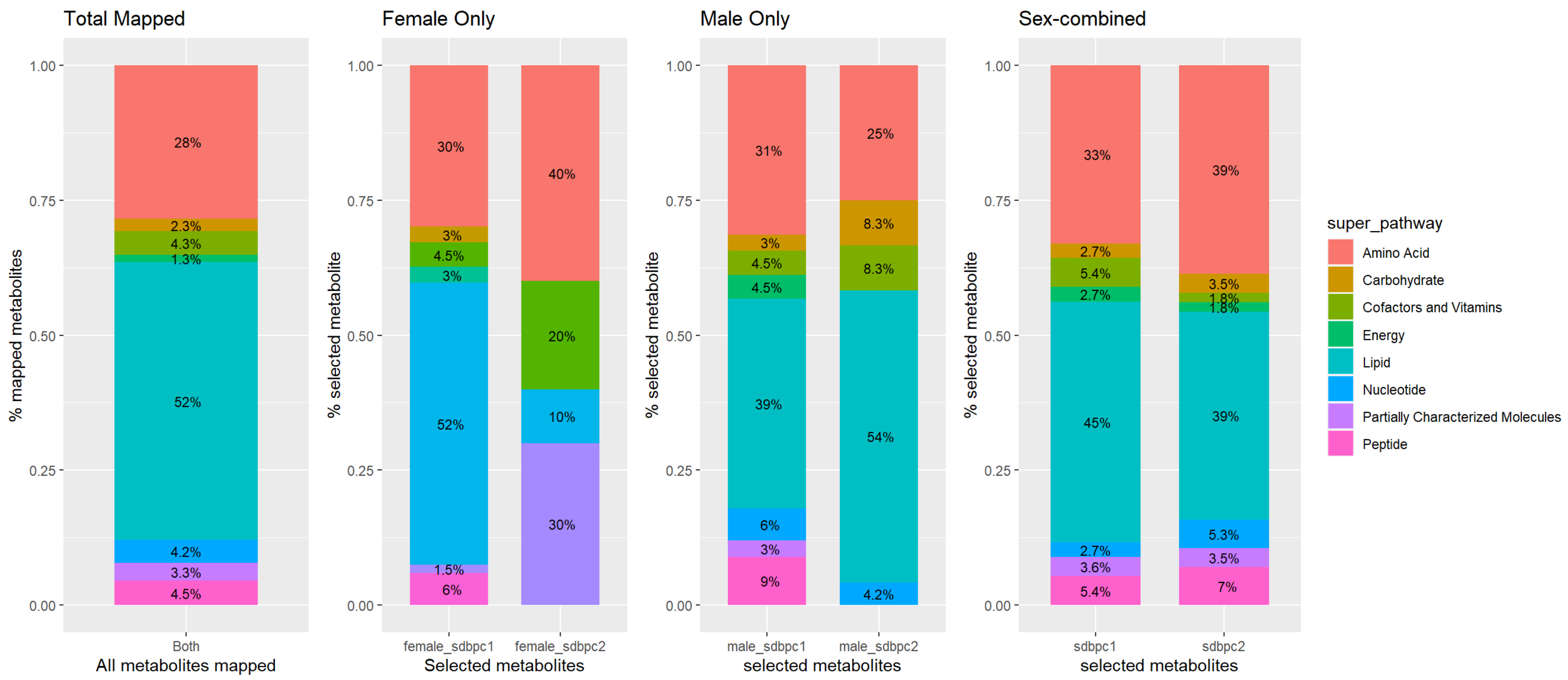

female_sdbpc1: metabolites selected by LASSO regression trained in females only to predict SDB PC1 in the discovery dataset (batch 1); female_sdbpc2: metabolites selected by LASSO regression trained in females only to predict SDB PC2 in the discovery dataset (batch 1); male_sdbpc1: metabolites selected by LASSO regression trained in males only to predict SDB PC1 in the discovery dataset (batch 1); male_sdbpc2: metabolites selected by LASSO regression trained in males only to predict SDB PC2 in the discovery dataset (batch 1); sdbpc1: metabolites selected by LASSO regression trained in both sexes combined to predict SDB PC1 in the discovery dataset (batch 1); sdbpc2: metabolites selected by LASSO regression trained in both sexes combined to predict SDB PC2 in the discovery dataset (batch 1);

Supplementary Figure S5. The associations between SDB metabolomic indices in quartiles and incident cardiometabolic outcomes in the combined batch in model1

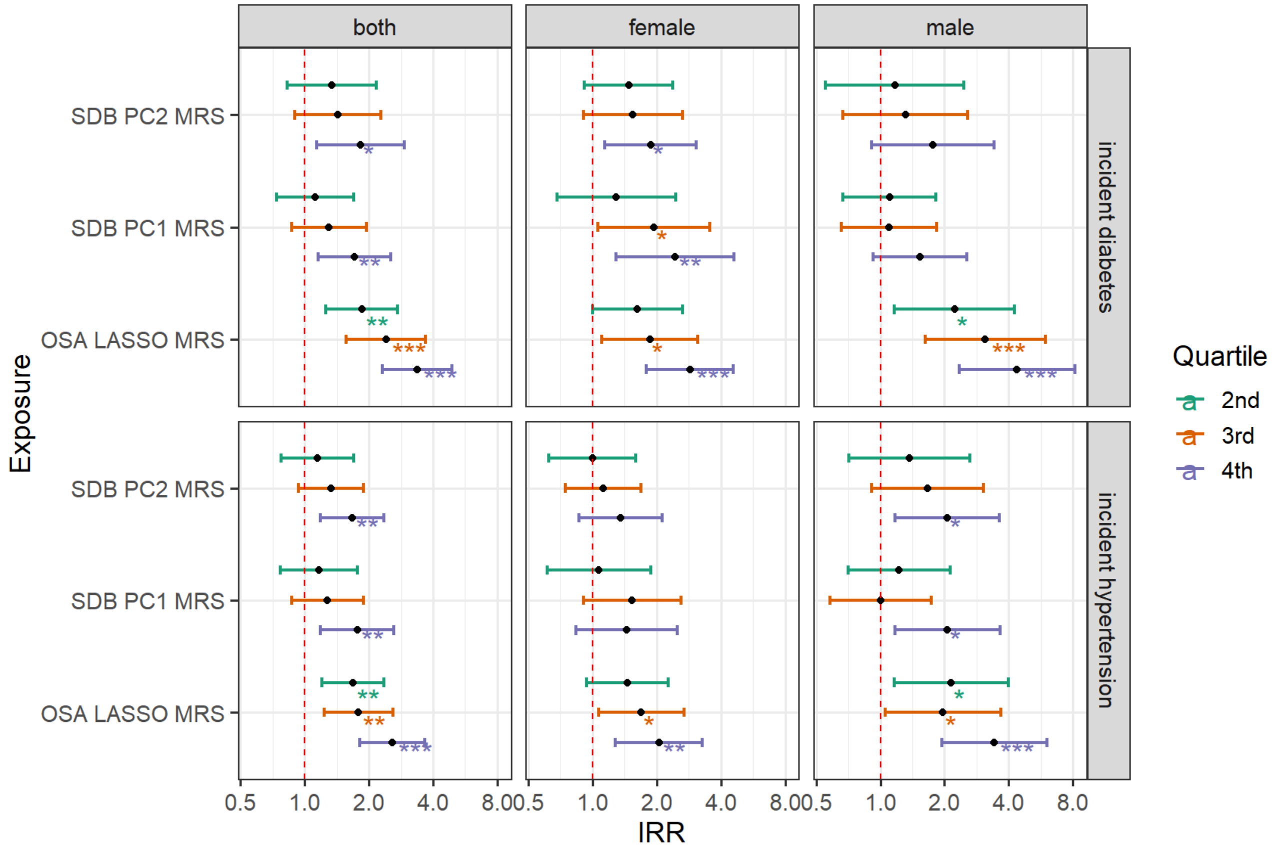

* indicates *p*<0.05. ** indicates *p*<0.01. *** indicates *p*<0.001. Incidence rate ratios (IRR) were calculated using survey-weighted generalized linear regressions for each pair of cardiometabolic outcome (i.e., incident diabetes, incident hypertension) and SDB phenotypes as the predictor in the combined dataset (batch 1 and batch 2), and are presented as effect estimates with 95% confidence intervals (CIs) for each quartile using the first quartile as the reference. *p* values were derived from a one degree-of-freedom Wald test. Exact IRR, 95% CIs and *p* values are provided in Supplementary Table S15. SDB PC1 MRS: metabolite risk score calculated based on the coefficients from LASSO regression trained in both sexes combined to predict SDB PC1 in the discovery dataset (batch 1); SDB PC2 MRS: metabolite risk score calculated based on the coefficients from LASSO regression trained in both sexes combined to predict SDB PC2 in the discovery dataset (batch 1); OSA LASSO MRS: metabolite risk score calculated based on coefficients from LASSO regression trained to predict OSA in previous publication (1). Incident diabetes is defined as fasting glucose >=126 mg/dL, or post-OGTT glucose >=200 mg/dL or A1C>=6.5%, or self-report of diabetes (n=2,908 samples); incident hypertension is defined as systolic or diastolic blood pressure is greater than or equal to 140/90 or participant self-reported as currently taking antihypertensive mediations (n=2,388 samples).

Supplementary Figure S6. Study sample selection for the metabolomic analysis

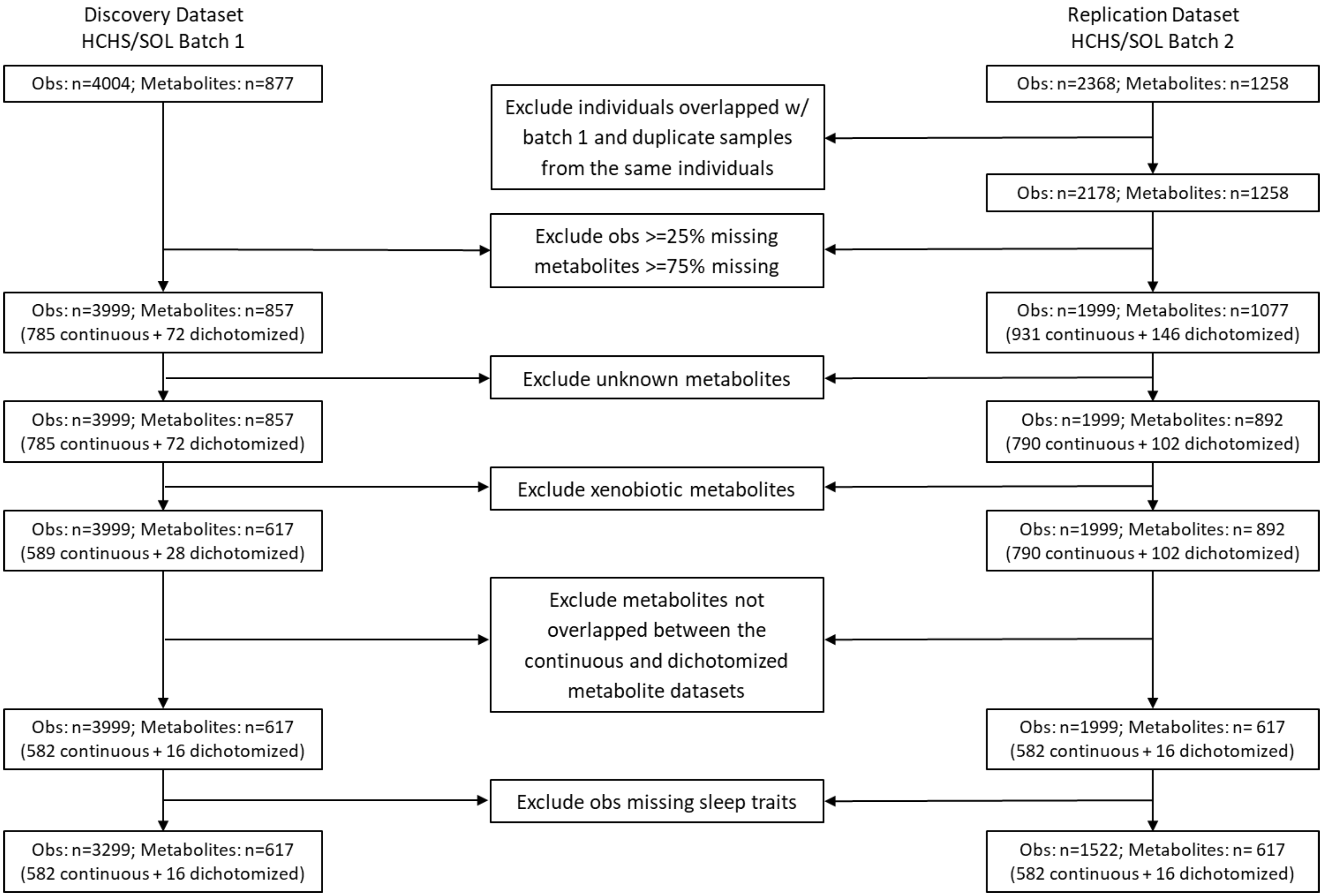

Obs: observations

References

1. Rodriguez CJ, Dharod A, Allison MA, Shah SJ, Hurwitz B, Bangdiwala SI, et al. Rationale and Design of the Echocardiographic Study of Hispanics/Latinos (ECHO-SOL). Ethn Dis. 2015;25(2):180–186.
