# Supplementary material for: Metabolomic profiles of sleep-disordered breathing are associated with hypertension and diabetes mellitus development
Source: Nat Commun. 2024 Feb 28;15:1845. doi: 10.1038/s41467-024-46019-y (PMC10902315; doi:10.1038/s41467-024-46019-y)
Supplement: Supplementary file 3 — Description of Additional Supplementary Files [file 41467_2024_46019_MOESM3_ESM.pdf]

Description of Additional Supplementary Files

File Name: Supplementary Data 1-18

Description: File with supplementary Data 1-18
