# Supplementary material for: Metabolomic profiles of sleep-disordered breathing are associated with hypertension and diabetes mellitus development
Source: Nat Commun. 2024 Feb 28;15:1845. doi: 10.1038/s41467-024-46019-y (PMC10902315; doi:10.1038/s41467-024-46019-y)
Supplement: Supplementary file 5 — Reporting Summary [file 41467_2024_46019_MOESM5_ESM.pdf]

nature portfolio

Corresponding author(s): Tamar Sofer

Last updated by author(s): Jan 30, 2024

Reporting Summary

Nature Portfolio wishes to improve the reproducibility of the work that we publish. This form provides structure for consistency and transparency in reporting. For further information on Nature Portfolio policies, see our Editorial Policies and the Editorial Policy Checklist.

Statistics

For all statistical analyses, confirm that the following items are present in the figure legend, table legend, main text, or Methods section.

n/a	Confirmed	
☐	☒	The exact sample size (*n*) for each experimental group/condition, given as a discrete number and unit of measurement
☐	☒	A statement on whether measurements were taken from distinct samples or whether the same sample was measured repeatedly
☐	☒	The statistical test(s) used AND whether they are one- or two-sided<br>*Only common tests should be described solely by name; describe more complex techniques in the Methods section.*
☐	☒	A description of all covariates tested
☐	☒	A description of any assumptions or corrections, such as tests of normality and adjustment for multiple comparisons
☐	☒	A full description of the statistical parameters including central tendency (e.g. means) or other basic estimates (e.g. regression coefficient) AND variation (e.g. standard deviation) or associated estimates of uncertainty (e.g. confidence intervals)
☐	☒	For null hypothesis testing, the test statistic (e.g. *F*, *t*, *r*) with confidence intervals, effect sizes, degrees of freedom and *P* value noted<br>*Give P values as exact values whenever suitable.*
☒	☐	For Bayesian analysis, information on the choice of priors and Markov chain Monte Carlo settings
☐	☒	For hierarchical and complex designs, identification of the appropriate level for tests and full reporting of outcomes
☐	☒	Estimates of effect sizes (e.g. Cohen's *d*, Pearson's *r*), indicating how they were calculated

Our web collection on statistics for biologists contains articles on many of the points above.

Software and code

Policy information about availability of computer code

Data collection	No data was collected as part of this manuscript.
Data analysis	All analyses were done in R 3.6.3. svyglm was used for survey-weighted generalized linear regression models, and svyprcomp was used for sampling-weighted principal component analysis, both of which were from the survey package (Lumley, T. Analysis of complex survey samples. J. Stat. Softw. 9, (2004)). The glmnet R package (version 3.0; Friedman, J., Hastie, T. & Tibshirani, R. Regularization Paths for Generalized Linear Models via Coordinate Descent. J. Stat. Softw. 33, 1–22 (2010)) was used for the LASSO linear regression. The code used for conducting the analyses, as well as for the generation of figures and tables presented in this study, are written in R, and available at https://github.com/yzhang104/HCHS-SOL_SDB_metabolomics.git (Zhang, Y. Metabolomic Profiles of Sleep-Disordered Breathing are Associated with Hypertension and Diabetes Mellitus Development: the HCHS/SOL. Zenodo (2024). doi:10.5281/zenodo.10558178)

For manuscripts utilizing custom algorithms or software that are central to the research but not yet described in published literature, software must be made available to editors and reviewers. We strongly encourage code deposition in a community repository (e.g. GitHub). See the Nature Portfolio guidelines for submitting code & software for further information.

Data

Policy information about availability of data

All manuscripts must include a data availability statement. This statement should provide the following information, where applicable:

- Accession codes, unique identifiers, or web links for publicly available datasets
- A description of any restrictions on data availability
- For clinical datasets or third party data, please ensure that the statement adheres to our policy

HCHS/SOL data are available through application to the data base of genotypes and phenotypes (dbGaP) accession phs000810. HCHS/SOL metabolomics data are available via data use agreement with the HCHS/SOL Data Coordinating Center (DCC) at the University of North Carolina at Chapel Hill, see collaborators website: https://sites.cscc.unc.edu/hchs/. Researchers can email the HCHS/SOL DCC at hchsadministration@unc.edu. The metabolite association data generated in this study are provided in the Supplementary Information. Source data for results presented in the figures are provided with this paper.

Research involving human participants, their data, or biological material

Policy information about studies with human participants or human data. See also policy information about sex, gender (identity/presentation), and sexual orientation and race, ethnicity and racism.

Reporting on sex and gender	Gender was self-reported according to a multiple choice question with "male" and "female" as potential responses, and was later verified as biological sex for all genotyped individuals.
Reporting on race, ethnicity, or other socially relevant groupings	All participants self-identified as Hispanic/Latino.
Population characteristics	Described in Table 1.
Recruitment	The Hispanic Community Health Study / Study of Latinos (HCHS/SOL) is a prospective community-based cohort study of 16,415 Hispanic/Latino individuals aged 18–74 years at the baseline examination (2008-2011)33. Individuals were selected into the study using a multi-stage stratified random sampling from four geographic regions: Bronx NY, Chicago IL, Miami FL, and San Diego CA.
Ethics oversight	The HCHS/SOL was approved by the institutional review boards (IRBs) at each field center, where all participants gave written informed consent, and by the Non-Biomedical IRB at the University of North Carolina at Chapel Hill, to the HCHS/SOL Data Coordinating Center. All IRBs approving the HCHS/SOL study are: Non-Biomedical IRB at the University of North Carolina at Chapel Hill. Chapel Hill, NC; Einstein IRB at the Albert Einstein College of Medicine of Yeshiva University. Bronx, NY; IRB at Office for the Protection of Research Subjects (OPRS), University of Illinois at Chicago. Chicago, IL; Human Subject Research Office, University of Miami. Miami, FL; Institutional Review Board of San Diego State University, San Diego, CA. All methods and analyses of HCHS/ SOL participants' materials and data were carried out in accordance with human subject research guidelines and regulations. This work was approved by the Mass General Brigham IRB and by the Beth Israel Deaconess Medical Center Committee on Clinical Investigations.

Note that full information on the approval of the study protocol must also be provided in the manuscript.

Field-specific reporting

Please select the one below that is the best fit for your research. If you are not sure, read the appropriate sections before making your selection.

☒ Life sciences ☐ Behavioural & social sciences ☐ Ecological, evolutionary & environmental sciences

For a reference copy of the document with all sections, see nature.com/documents/nr-reporting-summary-flat.pdf

Life sciences study design

All studies must disclose on these points even when the disclosure is negative.

Sample size	The main, batch 1, discovery dataset (used for SDB-SMA analysis and LASSO regression) included 1,874 female participants (mean age =42.8), and 1,425 male participants (mean age= 41.6), and the validation dataset included 960 female participants (mean age= 51.9) and 562 male participants (mean age=51.2) from batch 2. As this was a data analysis based on existing data, the sample size was determined based on data availability (i.e. the number of individuals available with appropriate data -- metabolomics and relevant phenotypes). Discovery and replication datasets were formed according to the separate assay batches of the metabolomics data, and so that no participants overlap between the datasets.
Data exclusions	Individuals were excluded if they did not have relevant data: sleep or metabolomics.
Replication	Our study included two "batches" of distinct samples from the same source population. Discovery of metabolite associations was performed on the first batch, and replication on batch 2. Five associations of single metabolite with a sleep disordered breathing measure were replicated. The associations of the two metabolite risk scores with their sleep disordered breathing measure replicated.
Randomization	This is an observational study, there was no randomization.

Blinding	No blinding, because it is irrelevant to this study design, which used unidentified data. No treatment was assigned and individuals could not be identified from the data.

Reporting for specific materials, systems and methods

We require information from authors about some types of materials, experimental systems and methods used in many studies. Here, indicate whether each material, system or method listed is relevant to your study. If you are not sure if a list item applies to your research, read the appropriate section before selecting a response.

Materials & experimental systems

n/a	Involved in the study
☒	☐ Antibodies
☒	☐ Eukaryotic cell lines
☒	☐ Palaeontology and archaeology
☒	☐ Animals and other organisms
☒	☐ Clinical data
☒	☐ Dual use research of concern
☒	☐ Plants

Methods

n/a	Involved in the study
☒	☐ ChIP-seq
☒	☐ Flow cytometry
☒	☐ MRI-based neuroimaging
